# The Origin and Invasion Pathway of Brown Rats *Rattus norvegicus* on Dok-Do Island Revealed by Genome-Wide Markers from 3-RADseq Approach

**DOI:** 10.3390/ani13071243

**Published:** 2023-04-03

**Authors:** Han-Na Kim, Ohsun Lee, Hwa-Jin Lee, Gyu-Cheol Kim, Hyeon-Soo Kim, Jonathan James Derbridge, Yeong-Seok Jo

**Affiliations:** 1 Department of Biology Education, Daegu University, Gyeongsan 38453, Republic of Korea; 2 School of Natural Resources and the Environment, University of Arizona, Tucson, AZ 85721, USA

**Keywords:** RADseq, brown rat, Dok-do, invasion pathway, island, Ulleung-do

## Abstract

**Simple Summary:**

Biological invasion on islands poses ecological threats, and understanding invasion pathways can aid in preventing invasive species. Recently, brown rats (*Rattus norvegicus*) arrived on Dok-do, a remote, small island in the East Sea of Korea. Due to strict regulation on human disturbances, including trapping, we adopted 3-RADseq to compensate for our small sample size and determine the origin of the invading rats. Our genomic analysis will aid in controlling and managing invasive species on this protected island.

**Abstract:**

Biological invasions are known to cause local extinctions on islands. Dok-do, a small, remote volcanic island in the East Sea of Korea in the western Pacific, has recently been invaded by rats, posing ecological problems. To infer their origin and invasion pathway, we collected rats from Dok-do and from the potential introduction source locations, Ulleung-do in the Pacific Ocean, and four east coastal ports. First, we identified that the brown rat (*Rattus norvegicus*) was the only rat species occurring at collecting sites based on the key morphological characteristics. To determine the population-level genetic diversity pattern, we applied the 3-RADseq approach. After a series of filtrations (minor allele frequency < 0.05, Hardy–Weinberg equilibrium *p* < 1 × 10^−7^), 4042 SNPs were retained for the final dataset from the 25,439 SNPs initially isolated. The spatial structure and genetic diversity pattern of brown rats suggested that the rat population on Dok-do was likely introduced from Ulleung-do. Our work provides practical information that will assist in the management of invasive brown rats in vulnerable island ecosystems.

## 1. Introduction

Island ecosystems are more vulnerable to ecological disturbance than mainland ecosystems [1], and small, remote islands have relatively high species extinction rates [2]. Non-native rodents, such as rats (*Rattus* spp.) and mice (*Mus* spp.), have been documented as key contributors to the extinction of at least 50 island species and negative effects on over 170 species across more than 40 islands [3]. After removing non-native rodents from New Zealand islands, invertebrates, reptiles, and birds have recovered completely [3]. Bird populations on islands are also known to increase following rodent removals [3,4,5].

The first anthropogenic introductions of *Rattus rattus* to Mediterranean islands likely occurred between B.C.E. 5550 and 8000 [6]. Followed by *R. rattus*, *R. norvegicus* has become the dominant rat on islands around Europe and East-South America since the 1700s [7]. For several centuries, however, there was nearly no effort to remove invasive rodents from island ecosystems. In 1951, *Rattus norvegicus* was extirpated from French Rouzic Island (3.3 ha), which was the first successful removal in history [8]. Since then, rodent removal has been implemented on more than 284 islands around the world (47,628 ha), and about 90% of projects have been successful [9].

The Korean Peninsula has 3348 adjunct islands, which provide important stop-overs for migratory birds [10]. Around the Korean Peninsula, most of the islands are located in the Yellow Sea, whereas only a few islands are located in the East Sea. Dok-do (island) is a lone island in the middle of the East Sea between Korea and Japan, such as the Hawaiian Islands in the Pacific, and therefore the island offers a crucial stop-over point for migratory birds passing the East Sea [11]. Dok-do is a volcanic island consisting of two islands, Dong-do (east islet) and Seo-do (west islet), with 89 small islets [12]. Irregular faunal surveys since after the Korean War and regular surveys since the 1900s have been conducted on Dok-do, and no sign of wild terrestrial mammals has been reported from this small volcanic island.

The first report of terrestrial mammals in Dok-do was 40 domestic rabbits (*Oryctolagus cuniculus domesticus*), which were released for food supply by the police in 1973, and the rabbit population increased to 230 rabbits [13]. Fortunately, the rabbit removal project was started by Ulleung-Gun (county) in 1988, and the rabbits were completely removed by 1992 [14]. No further terrestrial mammals were confirmed on Dok-do until 2008 [15]. 

In 2008, some construction workers observed a rodent in building materials on Seo-do, the west islet, and rodent signs were identified during the following year at the fisherman’s shelter on Seo-do [16]. The rodent signs were again reported from Dong-do, the east islet of Dok-do, in 2015 [17]. Although the rodents on the island were regarded as *Rattus* sp., even the species identification with voucher specimens was not properly conducted. Given the mixed distribution among the three rodent species, *Rattus norvegicus*, R. *tanezumi*, and *R. rattus* in Korea, identification of the rat species is almost impossible without a proper specimen. Since Ulleung-do, the closest island to Dok-do, had two *Rattus* spp. [18], the identity of rodents on Dok-do was not certain.

The primary purpose of this study was to identify the origin of the rats occurring on Dok-do. By employing a population genomics approach, we tried to infer the invasion path of the rat in Dok-do. The origin and invasion routes of the island populations will provide necessary information for the effective management of invasive species and the prevention of future immigration in Dok-do.

## 2. Methods and Materials

### 2.1. Study Sites

**Figure 1:**
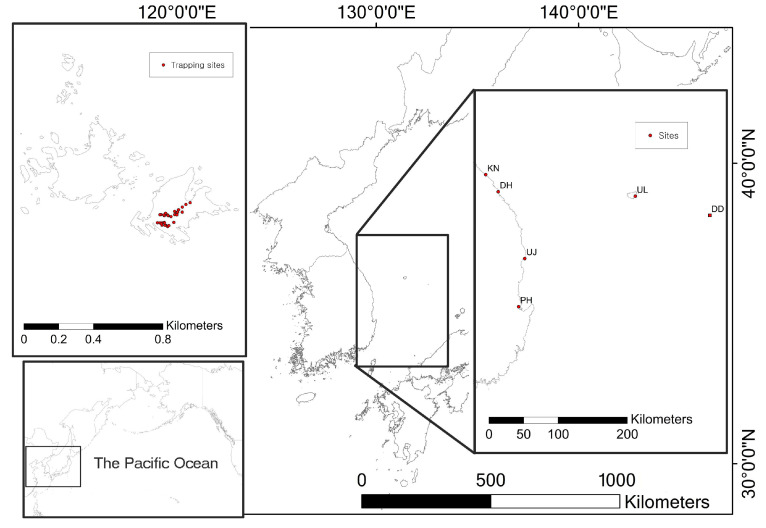
Trapping sites for rats (*Rattus* sp.). Dok-do (left above inset). Relative position of study area within the Pacific Ocean (left below inset). Korean Peninsula east coast and Ulleung-do with Dok-do (right inset). See Table 1 for abbreviation definitions.

Dok-do is the Korean Peninsula’s easternmost island, located 216.8 km from the mainland, in the western Pacific (Figure 1). Since the government did not permit entry to Seo-do, the study was conducted only on Dong-do. Dong-do is 73,297 m^2^ in size, and the highest peak is 98.6 m with a mean slope of 60° [19]. According to the automatic weather station, the annual mean temperature was 13.6 °C (highest temperature 32 °C and lowest temperature −8.5 °C) and the mean annual precipitation was 508 mm from November 2009 to January 2022. The island is rocky and sparsely vegetated. A platoon of police resides on the island. There are three daily arrival times for a tourist ferry, but tourists cannot freely travel from the pier area.

As the main gateway for Dok-do, Ulleung-do is the closest island (87.4 km from Dok-do, 130 km from the Korean Peninsula). The population of Ulleung-do was 8888 in 2021, and the mean number of annual visitors from 2001 to 2020 was 272,305 [20]. In Ulleung-do, four port areas were selected for trapping after conducting a preliminary rat survey. All traps were deployed around fishing net storage areas, where we found rat skulls and remains during the preliminary survey.

In addition to Dok-do and Ulleung-do, we also trapped rats from four ports that have regular ferries to Ulleung-do. Pohang (PH in Figure 1), a mainland city located 214 km from Ulleung-do, has two ports to Ulleung-do for passengers and cargo. We trapped small mammals from storage areas in the passenger terminal and food waste disposal area in the cargo terminal. Uljin (UJ in Figure 1), a mainland county located 144 km from Ulleung-do, has Hupo Port. A ferry departs from Hupo and arrives at Dok-do through Ulleung-do. This is the only ferry to Dok-do from inland. We trapped rats from garbage sites around the passenger terminal of Hupo Port. Mukho Port is located in Donghae (DH in Figure 1), a mainland city located 158 km from Ulleung-do. We deployed traps at the seawall where fishing nets were stacked. Kangneung Port in Kangneung City (KN in Figure 1), located 181 km from Ulleung-do, has restaurants around the passenger terminal. We trapped rats from food garbage disposal sites around restaurants. 

#### Sample Collection and DNA Extraction

We conducted sampling from six collection sites, including Dok-do, Ulleung-do, and four ports along the east coast (Figure 1). Due to the limited permission for entry into Dok-do, we only collected samples from the east islet of Dok-do, Dong-do. We deployed traps five times in areas of human disturbance, using 26 Sherman live traps in 2021 (a total of 2210 trap nights). 

On Ulleung-do, we conducted eight trapping sessions from November 2020 to July 2021, except for January 2021. We deployed 66 Sherman live traps, and the total trap nights were 2860. Most traps were deployed at port areas.

At four ports along the east coast, we deployed 10–20 commercial live rat traps for each port. Since Sherman live traps were lost at the first round of trapping, we used obvious commercial rat traps purchased from hardware stores. We conducted 14 one- to two-night trapping sessions (3 trapping sessions from October to December 2020 and 11 trapping sessions from January to July 2021) for each port.

We prepared a voucher specimen (i.e., a study skin and skull) from at least one sample from each collecting site. All voucher samples and tissues, along with the extracted DNA, were stored at the mammalogy laboratory at Daegu University. 

Genomic DNAs were extracted from the muscle tissues of 18 individuals using a QIAGEN blood and tissue kit (Qiagen, Valencia, CA, USA), following the manufacturer’s protocol. We inspected the quality of the isolated DNAs by visualizing the DNA on a 1% agarose gel through gel electrophoresis. 

### 2.2. 3-RADseq (Triple Enzyme Restriction-Site Associated DNA Sequencing)

For genotyping, we employed 3-RAD [21]. Using three restriction enzymes (two restriction enzymes and a third restriction enzyme to cut adapter dimers), 3-RAD has more efficiency than previous RAD approaches and improves the yield of the amplified reads [22]. For the 3-RAD library preparation, we applied three enzymes: EcolRI-HF and Xbal for digesting genomic DNA, and Nhel for cleaving dimers (all enzymes from Thermo Fisher Scientific, Waltham, MA, USA). We digested the DNA in a 15 μL reaction mixture containing 100 ng DNA, 8 μL master mix, and 1 μL of adapter (5 μM). The master mix consisted of 1 μL of three enzymes, 1.5 μL of 10× FastDigest Buffer, and 3.51 μL of ultra-pure water. The mixture was incubated for 15 min at 37 °C. Subsequently, we prepared the ligation mixture with 0.5 μL of ligase buffer, 100 units of T4 DNA ligase, 1.5 μL of 10 mM ATP, and 2 μL of ultra-pure water. We added 5 μL of ligation mixture to each digested sample. The digestion and ligation were then administered simultaneously. Additional incubation was conducted at 22 °C for 20 min and 37 °C for 10 min, and the incubation process was repeated twice. We inactivated the enzymes by the final incubation step at 80 °C for another 20 min.

To check the ligation status, we performed a test PCR using a Bioneer multiplex premix. We prepared a 20 μL reaction mixture containing 1 μL of each of the iTru5 and iTru7 primers and 1 μL of adaptor-ligated DNA fragments. The test PCR profile was 95 °C for 10 min; 35 cycles of 95 °C for 30 s, 60 °C for 1 min, 72 °C for 30 s; and 72 °C for 5 min. Then, we pooled 5 μL of the adaptor-ligated fragments and purified them using a 1:1.8 mixture of AmPure XP magnetic beads (Beckman Coulter, Brea, CA, USA).

Samples were washed with 70% EtOH and resuspended in 60 μL of ultra-pure water. For PCR enrichment, we prepared 50 μL of a reaction mixture containing 5 μL of the pooled DNA fragments, 5 μL of 5 μM iTru5 primer, 5 μL of 5 μM iTru7 primer, 10 μL of 5× HF buffer, 1.5 μL of 10 μM dNTP, and 1 unit of Phusion DNA polymerase. The amplification condition was 98 °C for 2 min; 7 cycles of 98 °C for 20 s, 60 °C for 15 s, 72 °C for 30 s; and 72 °C for 5 min. Sample clean-up was followed by the same procedure of test PCR purification. 

Since we targeted 400 bp fragments, we used the Pippin Prep (Sage Science, Beverly, MA, USA) for size selection. The purified samples on a 2% agarose cassette were processed in the Pippin Prep under 400 bp tight conditions. 

For adjusting concentrations, we conducted additional amplification with 50 μL of reaction mixture consisting of 5 μL of 400 bp DNA fragments, 3 μL of 5 μM P5 primer, 3 μL of 5 μM P7 primer, 1.5 μL of 10 μM dNTP, 10 μL of 5× HF buffer, and 1 unit of Phusion DNA Polymerase. The final PCR profile was 12 cycles of 98 °C for 2 min, 98 °C for 20 s, 61 °C for 15 s, 72 °C for 45 s, and 72 °C for 5 min. The final clean-up was the same for the test PCR and PCR enrichment. 

To check the quality and quantity of the completed 3-RAD library (clear peak of 400 bp ± 50 and >5 nM), we evaluated the samples using an Agilent 2100 Bioanalyzer (Agilent Technologies, Santa Clara, CA, USA). Then, we sent the samples to Macrogen Inc., Seoul, Republic of Korea, for running the library on an Illumina HiSeq X-10 (LAS Inc., North Palm Beach, FL, USA) using 2 × 150 paired-end sequencing.

### 2.3. Genomic Data Analysis

All sequenced raw data were processed in Stacks version 2.41 [23]. We excluded low-quality reads using the process_tag function based on a Phred score of 10. 

RAD loci were assembled de novo for fast processing. The parameters for demultiplexing and adapter trimming were -n3, -m3, and -M3 [24]. The RAD catalog assembled by the process_tag function was applied to SNP matrix construction. SNPs were called on a population function implemented in Stacks when the catalogs were constructed. We extracted the SNP loci if the loci were present in at least 80% of the samples within each population and shared by at least 5 populations (−p 5 and −r 0.8). Only the first SNP per locus (-write-single-snp) was selected to ensure the independence of SNP loci, avoiding linkage disequilibrium (LD). We then filtered SNP loci based on Hardy–Weinberg Equilibrium (HWE, threshold = *p* < 10 × 10^−6^; Li, 2011; [25]) to avoid the loci with extreme heterozygosity and assembly errors in Plink ver. 1.9 [26]. Finally, we purged genotypes with more than 30% missing calls and SNP loci with a minor allele frequency of ≤0.05 in Tassel 5.0 [27]. 

With the finalized 4042 SNPs, we computed the following diversity parameters: the number of alleles (*Na*), the effective number of alleles (*Ne*), Shannon’s information index (*I*), and expected heterozygosity (*He*), using GenAlex 6.5 [28]. For the population divergence, we calculated pairwise F*_ST_* using Arlequin version 3.5 with 1000 permutations for the significance test [29]. 

To examine cluster assignment, we employed two approaches: principal coordinate analysis (PCoA) and Bayesian model-based clustering. PCoA was performed by GenAlex 6.5 (The Australian National University, Canberra, Australia) (Available online: https://biology-assets.anu.edu.au/GenAlEx/Welcome.html accessed on 2 April 2023) based on Nei’s genetic distance. For Bayesian clustering, we employed STRUCTURE v. 2.3.4 [30] and tested K = 1 to K = 6 and repeated 5 runs for each K with 100,000 burn-in steps and 1,000,000 MCMC iterations. The admixture of the ancestry model and the correlated allele frequency model was used for the STRUCRUTE analysis [31]. To infer the optimal K, we used STRUCTURE HARVESTER Web v. 0.6.94 [32] and calculated ΔK based on Evanno et al. [33]. 

## 3. Results

A total of 18 brown rats (*Rattus norvegicus*) were caught, with 5 from Dok-do, 3 from Ulleung-do, and 9 from the east coastal ports. However, one sample from Ulleung-do was excluded due to inappropriate DNA quality for Illumina sequencing. The 3-RAD library of the remaining 17 samples produced a total of 135,090,723,353 bp of 894,640,552 reads with a GC content of 44.92%. The number of DNA reads obtained for each sample ranged from at least 17,000,000 to a maximum of 150,000,000 (Supplementary Figure S1). However, one individual was removed from further analysis due to an excessive missing rate during the data filtration process. We initially isolated 25,439 SNPs from the SNP matrix and finally retained 4042 SNPs from 16 individuals for the downstream analysis after the pruning steps. 

The genetic diversity of *Na* adjusted by sample size in *R. norvegicus* was highest in Donghae (DH) but lowest in Uljin (UJ), with 1.848 and 1.206, respectively (Table 1). The genetic diversity of Dok-do was relatively well maintained (*Na* = 1.605, *He* = 0.27) compared to the port populations (UJ, PH, and KN). 

Pairwise F*_ST_* was the highest between Ulleung-do (UL) and Uljin (UJ), whereas Pohang (PH) and Donghae (DH) showed the lowest F*_ST_* (Table 2). The F*_ST_* value between Dok-do (DD) was the lowest for the population pair with DH, whereas it was the highest for the population pair with UJ. 

The top three principal components (PC) explained 17.76%, 9.97%, and 8.83% of the genetic variance. In the PC 1 and PC 2 graph, DD was the most closely clustered to UL, followed by PH, UJ, KN, and DH. On the plot based on PC 1 and PC 3, we observed a similar pattern but a more dispersed pattern along the third PC axis (Supplementary Figures S2 and S3). 

**Figure 2:**
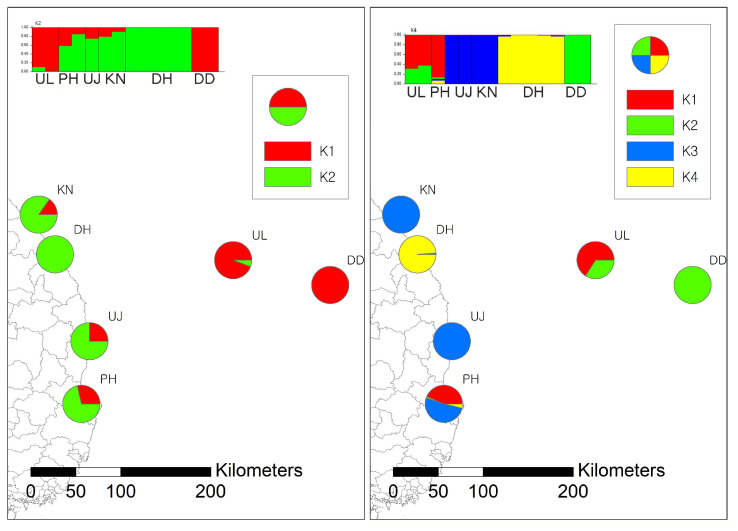
Spatial structure of 6 populations of *Rattus norvegicus* along the east coast with Ulleung-do and Dok-do. The bar and pie chart of clustering assignment for K = 2 (**left**) and 4 (**right**) assessed from STRUCTURE based on 4042 SNPs.

The △K was highest for the number of clusters, K = 2, followed by K = 4 (Figure 2). K = 2 and K = 4 showed a similar ancestry pattern for the Dok-do population. In both scenarios, the UL population shows shared ancestry with DD and PH (Figure 2 and Figure S4). In the K = 4 scenario, the DH population exhibited a unique ancestry pattern. In the K = 2 scenario, DD shares ancestry with UL, PH, UJ, and KN, whereas in the K = 4 scenario, DD shares ancestry only with UL.

## 4. Discussion

For centuries, anthropogenic species introductions have resulted in widespread negative effects on the biodiversity of native fauna, including the extinction of endemic species in island ecosystems [34]. During the 20th century, Dok-do has already experienced the extinction of the sealion *Zalophus japonicus* [10]. The island is protected by the South Korean government as a national monument and special protected area, but tourism has been allowed on the part of the island, Dong-do. Coupled with the periodic visits by the tourists, residing police and frequent activities of governmental agencies could pose ecological problems on the island. 

*Rattus norvegicus* is the most common rat in South Korea [10]. Around pier and port areas, however, *R. rattus* or *R. tanezumi* have been reported more commonly than *R. norvegicus* [35]. Through this study, based on the morphological characteristics, e.g., tail and ear lengths, we confirmed that the introduced rodents on Dok-do are *R. norvegicus*. It is also confirmed by the mitochondrial DNA barcode [36]. The prepared voucher specimens with tissue and DNA are stored at the mammalogy laboratory at Daegu University (#MM202102-MM202115). Except for *R. norvegicus*, we did not find any other terrestrial mammals on the island. 

Large sample sizes (20–30) are often used for population differentiation measures when the marker numbers are limited [37]. However, in some cases (e.g., studies on endangered or geographically restricted species), it is not always possible to obtain a large sample size. Our study system was a small island recently invaded by rats. We conducted an intensive trapping effort to collect as many samples as possible for a year (>6000 trap nights for this study), but the sampling source was very limited due to the nature of the species invasion history. Accordingly, we employed a genome-wide marker development approach to adjust the probable biases caused by the small sample size, as suggested by Willing et al. [38]. They showed that population divergence estimation such as F*_ST_* can be accurate and unbiased when a large number of bi-allelic markers such as SNPs are used for a small number of samples (*n* = 2, 4, 6).

According to the spatial genetic structure of K = 2 and K = 4, *R. norvegicus* on Dok-do is likely to have originated from Ulleung-do rather than from the east coastal regions. Another possible scenario could be a two-step invasion process, first from the coast to Ulleung-do and then from Ulleung-do to Dok-do. Since the number of alleles found in Dok-do is similar to those found in Ulleung-do, a two-step invasion process is plausible. In either case, a direct invasion from the mainland to Dok-do is implausible. 

Although K = 2 showed the highest △K value, K = 4 also showed a comparable △K value. In both K = 2 and K = 4 structures, it appears that initially a small founding population derived from the east coastal populations initially arrived in UL and has become the current UL population. When founding individuals immigrated to UL, many alleles, e.g., alleles assigned to the ancestry represented by green (K = 2) and blue (K = 4), were lost due to the founder effect (Figure 2). A similar founding process might have been repeated when the DD population was established. As the UL population has been established successfully, some of the UL individuals have started to disperse to the DD island and establish a population. When a small number of selected individuals transferred to Dok-do island from UL populations, it is highly likely that many alleles were lost, resulting in the current structure pattern. However, our results should be interpreted cautiously, since the data we collected are very limited due to the challenges involved in trapping these wild animals. For example, the DH population showed a unique pattern of ancestry in K = 4, yet the population is not geographically isolated, and there is no specific barrier to the immigration of *R. norvegicus*. The pattern of unique ancestry in DH is probably caused by limited sampling. Since there is a lack of samples from the neighboring populations, we cannot confirm whether the ancestry of DH is unique and not shared by any populations. 

Through the study, we determined that the origin of the DD population is the UL population, likely resulting from the frequent visits by tourism ships from Ulleung-do to Dok-do. Due to frequent visits by ships of various origins, an island such as Dok-do is always vulnerable to invasive animals or plants. To reduce species invasion via the mainland, thorough quarantine measures for every vessel are necessary. We are convinced that identifying the sources of non-native species is essential to any strategy aimed at controlling and preventing invasive species on islands.

## 5. Conclusions

The introduction of non-native species by humans has had devastating effects on native biodiversity, especially in island ecosystems. Our study highlights the vulnerability of islands such as Dok-do to invasive species and the importance of identifying the sources of non-native species to develop effective control and prevention strategies. Thorough quarantine measures for vessels may help reduce the risk of species invasion from the mainland.

## Figures and Tables

**Table 1:** Summary of genetic diversity indices for *Rattus norvegicus* along the east coast with Ulleung and Dok-do islands in South Korea. *Na*—number of alleles. *Ne*—number of effective alleles. *I*—Shannon’s information index. *He*—expected heterozygosity. The parentheses represent standard errors.

Population	UL (Ulleung)	PH (Pohang)	UJ (Uljin)	KN (Kangneung)	DH (Donghae)	DD (Dok-do)
The number of individuals	2	2	1	2	5	4
*Na*	1.501 (±0.008)	1.595 (±0.008)	1.206 (±0.006)	1.522 (±0.008)	1.848 (±0.006)	1.605 (±0.008)
*Ne*	1.501 (±0.008)	1.595 (±0.008)	1.206 (±0.006)	1.522 (±0.008)	1.675 (±0.006)	1.503 (±0.007)
*I*	0.348 (±0.005)	0.412 (±0.005)	0.143 (±0.004)	0.362 (±0.005)	0.528 (±0.004)	0.386 (±0.005)
*He*	0.251 (±0.004)	0.297 (±0.004)	0.103 (±0.003)	0.261 (±0.004)	0.367 (±0.003)	0.270 (±0.004)

**Table 2:** Mean pairwise F*_ST_* values were computed from 4042 SNPs among 6 populations of *Rattus norvegicus* on the east coast with Ulleung and Dok-do islands, Republic of Korea. See Table 1 for the population abbreviations. All values are statistically significant at *p* < 0.01.

	UL	PH	UJ	KN	DH	DD
UL	-					
PH	0.280	-				
UJ	0.458	0.398	-			
KN	0.324	0.269	0.429	-		
DH	0.251	0.191	0.318	0.214	-	
DD	0.281	0.286	0.441	0.322	0.242	-

## Data Availability

All sequences have been deposited in GenBank, project id SAMN34037271.

## Supplementary Materials

The following supporting information can be downloaded at: https://www.mdpi.com/article/10.3390/ani13071243/s1, Figure S1: Histogram of DNA reads number of 17 *Rattus norvegicus* from four ports along the east coast of Korea, Ulleung-do, and Dok-do; Figure S2: Principal component analysis (PCoA) plot for 16 *Rattus norvegicus* from four ports along the east coast of Korea, Ulleung-do, and Dok-do. PC1 and PC2 were plotted; Figure S3: Principal component analysis (PCoA) plot for 16 *Rattus norvegicus* from four ports along the east coast of Korea, Ulleung-do, and Dok-do. PC1 and PC3 were plotted; Figure S4: Delta K (ΔK) graph obtained by Structure Harvester.
