# Supplementary material for: The Origin and Invasion Pathway of Brown Rats Rattus norvegicus on Dok-Do Island Revealed by Genome-Wide Markers from 3-RADseq Approach
Source: Animals (Basel). 2023 Apr 3;13(7):1243. doi: 10.3390/ani13071243 (PMC10093337; doi:10.3390/ani13071243)
Supplement: Supplementary file 1 [file animals-13-01243-s001.zip › animals-2131314-supplementary.pdf]

animals

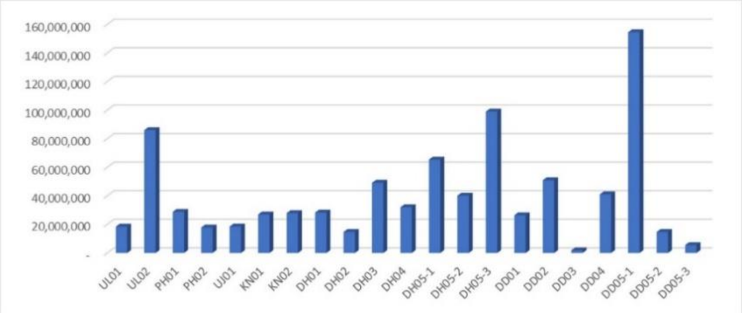

Supplementary Figure S1. Histogram of DNA reads number of 17 *Rattus norvegicus* from four ports along the east coast of Korea, Ulleung-do, and Dok-do.

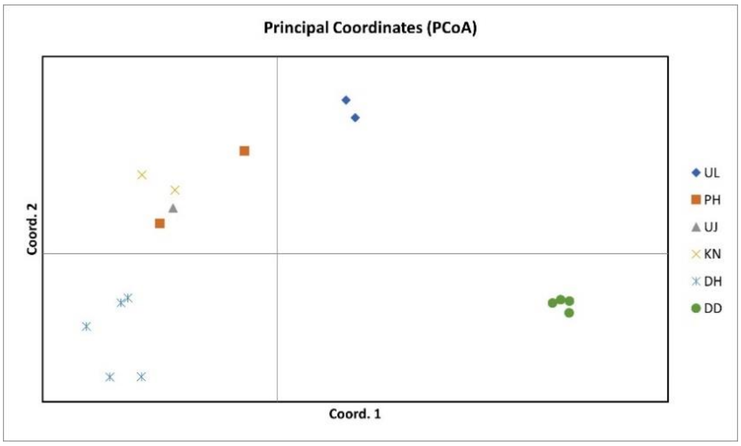

Supplementary Figure S2. Principal component analysis (PCoA) plot for16 *Rattus norvegicus* from four ports along the east coast of Korea, Ulleung-do, and Dok-do. PC1 and PC2 were plotted.

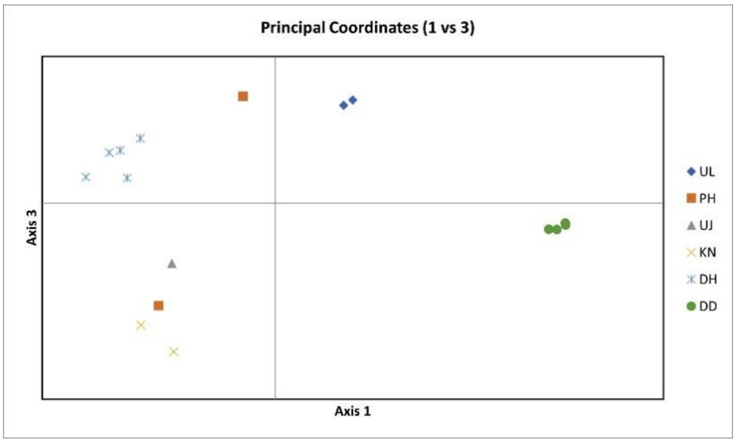

Supplementary Figure S3. Principal component analysis (PCoA) plot for 16 *Rattus norvegicus* from four ports along the east coast of Korea, Ulleung-do, and Dok-do. PC1 and PC3 were plotted. .

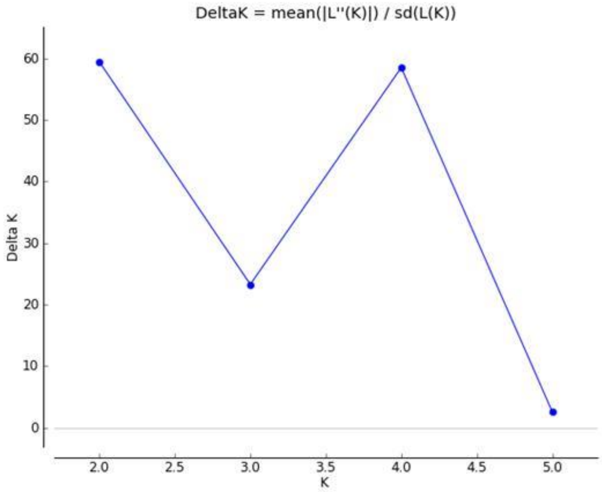

Supplementary Figure S4. Delta K (ΔK) graph obtained by Structure Harvester.
